# Transcriptome Analysis on Single Small Yellow Follicles Reveals That *Wnt4* Is Involved in Chicken Follicle Selection

**DOI:** 10.3389/fendo.2017.00317

**Published:** 2017-11-15

**Authors:** Yiya Wang, Qiuyue Chen, Zemin Liu, Xiaoli Guo, Yanzhi Du, Zhenjie Yuan, Miao Guo, Li Kang, Yi Sun, Yunliang Jiang

**Affiliations:** ^1^Shandong Provincial Key Laboratory of Animal Biotechnology and Disease Control and Prevention, College of Animal Science and Veterinary Medicine, Shandong Agricultural University, Tai’an, China

**Keywords:** chicken, follicle selection, follicle-stimulating hormone, granulosa cells, *Wnt4*

## Abstract

Ovarian follicle selection is an important process impacting the laying performance and fecundity of hens, and is regulated by follicle-stimulating hormone (FSH) through binding to its receptor [follicle-stimulating hormone receptor (FSHR)]. In laying hens, the small yellow follicle (6–8 mm in diameter) with the highest expression of FSHR will be recruited into the preovulatory hierarchy during ovarian follicle development. The study of molecular mechanism of chicken follicle selection is helpful for the identification of genes underlying egg-laying traits in chicken and other poultry species. Herein, the transcriptomes of chicken small yellow follicles differing in the mRNA expression of *FSHR* were compared, and a total of 17,993 genes were identified in 3 pairs of small yellow follicles. The Wnt signaling pathway was significantly enriched in the follicles with the greatest fold change in *FSHR* expression. In this pathway, the expression level of *Wnt4* mRNA was significantly upregulated with a log_2_(fold change) of 2.12. We further investigated the expression, function, and regulation of *Wnt4* during chicken follicle selection and found that *Wnt4* mRNA reached its peak in small yellow follicles; *Wnt4* stimulated the proliferation of follicular granulosa cells (GCs), increased the expression of *StAR* and *CYP11A1* mRNA in prehierarchical and hierarchical follicles, increased the expression of *FSHR* mRNA, and decreased the expression of anti-Müllerian hormone and *OCLN* mRNA. Treatment with FSH significantly increased *Wnt4* expression in GCs. Moreover, *Wnt4* facilitated the effects of FSH on the production of progesterone (P4) and the mRNA expression of steroidogenic enzyme genes in the GCs of hierarchical follicles, but inhibited the effects of FSH in the GCs of prehierarchical follicles. Collectively, these data suggest that *Wnt4* plays an important role in chicken follicle selection by stimulating GC proliferation and steroidogenesis. This study provides a theoretical basis for improving the egg-laying performance of chicken and a reference for the elucidation of the molecular mechanism of follicular selection in mammals.

## Introduction

The chicken ovary is a dynamic organ and a key component of the female reproductive system. Follicles of various sizes exist in the sexually mature chicken ovary and these can be broadly divided into prehierarchical follicles and hierarchical follicles (F1–F5) (1). During the laying period, follicles will be recruited into the preovulatory hierarchy from a cohort of small yellow follicles of 6–8 mm in diameter approximately once a day, a process termed follicle selection. The selected follicle will undergo rapid development from an F5 follicle to an F1 follicle until ovulation (2). This process is regulated by the hypothalamic–pituitary axis and the paracrine and autocrine factors from ovarian follicles (3). It is estimated that there are approximately 12,000 oocytes in a sexually mature hen, but only a few hundred of them are selected to mature and reach the ovulation stage (4); most small yellow follicles are not selected and undergo atresia (5, 6).

Follicle selection is indispensable in the reproductive process in female chickens, the hallmark of which is the proliferation and differentiation of granulosa cells (GCs). Several factors regulating the proliferation and differentiation of GCs have been reported, most of which belong to the transforming growth factor superfamily, for instance the group of bone morphogenetic proteins (BMPs) (7–9). In addition, anti-Müllerian hormone (*AMH*) could decrease follicle-stimulating hormone (FSH) sensitivity and regulate follicle selection in mouse GCs (10), is highly expressed in chicken small follicles, prior to follicle selection (11). Occludin (*OCLN)* is expressed in GCs and affects the process of yolk transport around the time of chicken follicle selection (12). In chicken follicular GCs, the expression of steroidogenic acute regulatory protein (*StAR*) and cytochrome P450 family 11 subfamily A member 1 (*CYP11A1)* is prerequisite for progesterone synthesis, and is related to follicle selection (13). Among the multiple small yellow follicles isolated from a single chicken ovary, one typically exhibits a higher expression level of follicle-stimulating hormone receptor (FSHR) compared to the other follicles (14) and thereby acquires responsiveness to FSH, resulting in the proliferation and differentiation of GCs. Other inhibitory or non-activated signals in the prehierarchical follicles of laying hens may also exist to prevent GCs from responding to FSH. However, the signal(s) that initiate FSH responsiveness within the selected follicles and the factors that trigger the differentiation of GCs in the process of follicle selection have not been clearly characterized.

Members of the Wnt family are secreted glycoproteins that were recently identified as regulators of ovarian function, acting as signaling molecules to modulate follicular responses to gonadotropins. Wnt proteins may act through β-catenin-dependent or β-catenin-independent pathways and their abnormal expression and activation may cause tumors (15–17). The β-catenin-dependent pathway is involved in the regulation of cell proliferation, cell fate determination, and embryonic induction (18, 19). Wnt signaling pathways are critical for ovarian development and essential for normal follicle development (20). The number of oocytes was found to be decreased in *Wnt4* (Wnt family member 4)-null female mice (21). *Wnt4* regulates the vascular boundary of the ovary in mice by regulating follistatin and maintains the survival of germ cells (22), and *Wnt4* conditional knockout mice displayed low fertility and blocked antral follicle development (20). In rodents, *Wnt4* was found to be specifically expressed in the follicular GCs and corpora lutea, and its expression level could be regulated by an ovulatory dose of hCG (21). In chickens, *Wnt4* was found to be highly expressed in the shell gland and isthmus of the oviduct and regulated by estrogen (23), but its role in follicular selection was not investigated.

To uncover the molecular mechanisms of follicle selection in hens, we identified the differentially expressed genes (DEGs) in small yellow follicles differing in *FSHR* expression by using Illumina RNA deep sequencing. A total of 1,999 DEGs were identified from the RNA-seq data. Among them, the mRNA expression level of *Wnt4* increased along with the upregulation of *FSHR* mRNA. Given the important role of FSH signaling in follicle selection, we hypothesized that the expression of *Wnt4* is likely regulated by FSH and *Wnt4* plays some roles in this process. In this study, we analyzed the expression dynamics of *Wnt4* in different-sized follicles, its effect on GC proliferation and differentiation and the effect of FSH on its expression in GCs of prehierarchical and hierarchical follicles. These results may help us to further understand the molecular mechanisms of follicle selection in hens.

## Materials and Methods

### Animals and Samples

Several sexually mature (30 weeks old) Jining Bairi hens, an indigenous chicken breed that is well known for early sexual maturity at around 100 days, were randomly selected from the local research farm affiliated with Shandong Agricultural University. All chickens had free access to water and feed. The chickens were housed in separate cages with a daily light period of 14 h and the laying events were monitored to determine the regular laying sequence. Three hens in the middle of a laying sequence were sacrificed by decapitation immediately after oviposition. All of the small yellow follicles (6–8 mm in diameter) were collected and stored in liquid nitrogen for RNA isolation. All of the animal experiments were approved by the Institutional Animal Care and Use Ethics Committee of Shandong Agricultural University and performed in accordance with the “Guidelines for Experimental Animals” of the Ministry of Science and Technology of China.

### RNA Isolation and RNA-Seq Library Preparation

Total RNA was extracted from all of the small yellow follicles (6–8 mm) using TRIzol reagent (Invitrogen) according to the manufacturer’s instructions. RNA purification, cDNA synthesis, and RNA-seq were performed at Biomarker Technologies Co., Ltd. (Beijing, China). Enrichment of eukaryotic mRNA was performed using a NEBNext Poly(A) mRNA Magnetic Isolation Module (NEB, E7490), and the rRNA and concentrated prokaryotic mRNA were removed using a MICROBExpress Bacterial mRNA Enrichment Kit (Invitrogen, AM1905). mRNA was used as a template to construct the library. The qualified library was clustered on an Illumina cBot and finally sequenced using an Illumina HiSeq 2500. Prior to analysis, the low-quality reads and rRNA sequences were removed, and the clean reads were mapped to the chicken genome. The structure and expression of DEGs were analyzed and the functional annotation of the genes was accomplished by comparison with the reference genome: *Gallus gallus* (assembly Gallus_gallus-5.0).

### Protein–Protein Interaction (PPI) Networks

Protein–protein interaction networks were constructed based on the information from STRING version 10.0, which permits the critical assessment and integration of PPIs, including both direct (physical) and indirect (functional) associations. Credible interactions (combined_score ≥0.4) were accepted for further network analysis using CytoScape.

### Cell Culture

The hierarchical follicles and all of the small yellow follicles were isolated from egg-laying hens and placed in phosphate-buffered saline (PBS). The small yellow follicles were treated with 0.1% collagenase II (MP Biomedicals, Santa Ana, CA, USA) at 37°C for 10 min to obtain the GCs. The yolks of the follicles were removed carefully with ophthalmic forceps and the GCs were isolated from the hierarchical follicles and then dispersed by treatment with 0.25% trypsin–EDTA (Gibco) at 37°C for 15 min with gentle oscillation in a centrifuge tube. After centrifugation, the GCs were suspended in culture medium (M199 with 10% fetal bovine serum and 1% penicillin/streptomycin), and subsequently seeded in 24-well culture plates at a density of 1 × 10^5^/well. The number of cells was detected using Trypan blue. Cells were cultured at 38°C in an atmosphere of water-saturated 5% CO_2_ for 24 h.

### Plasmid Construction and Cell Transfection

The coding DNA sequence region of *Wnt4* was cloned from chicken cDNA by PCR. The amplified fragment spanned the region between the transcription initiation site and the termination codon. PCR products were cloned into the pUSEamp vector (Millipore) to generate the overexpression plasmid. All of the primers are listed in Table S1 in Supplementary Material. The shRNA of *Wnt4* was synthesized by Shanghai GenePharma Co., Ltd. (Shanghai, China), and the target sequences are listed in Table S2 in Supplementary Material. GCs were plated on 24-well plates for the transient transfection experiments. The cells were transfected with the overexpression vector and shRNA (800 ng/well) using Lipofectamine LTX and Plus Reagent (Invitrogen). Six hours after transfection, recombinant FSH was added. Twenty-four hours after transfection, the cells were lysed for RNA and protein extraction.

### Real-time Quantitative PCR

The total RNA was extracted from all of the follicles using TRIzol reagent (Invitrogen), and the total RNA from the cultured GCs was isolated using a MicroElute Total RNA Kit (Omega). The quality of the total RNA samples was tested by gel electrophoresis and spectrophotometry. The cDNA was synthesized using a PrimeScript RT Reagent Kit with a gDNA Eraser (TaKaRa, Dalian, China). The total reaction volume was 20 µL, containing 1 µg total RNA, 1 µL oligo(dT)_18_ primer, 1 µL of PrimeScript RT Enzyme Mix I, 4 µL of 5× PrimeScript Buffer, and RNase-free ddH_2_O. The program was 37°C for 15 min and 85°C for 5 s. Real-time quantitative PCR was performed on an MX3000p system (Stratagene, La Jolla, CA, USA). The reaction system contained 1.5 µL cDNA, 7 µL of 2× SYBR Premix Ex Taq II (TaKaRa, Dalian, China), 0.3 µL of 50× Rox Reference Dye II, 0.3 µL of each of the forward and reverse primers, and RNase-free ddH_2_O. The program was 95°C for 30 s, and 40 cycles of 95°C for 5 s, 60°C for 30 s, and 72°C for 20 s. Melting curves were used to confirm the specificity of each product, and the PCR efficiency was determined by analysis of two-fold serial dilutions of the cDNA. The PCR efficiency was close to 100%, allowing the use of the 2^−ΔΔCT^ method for the calculation of relative gene expression (24). The mRNA expression of the genes *FSHR, Wnt4, StAR, CYP11A1, AMH, OCLN*, and so on was quantitatively analyzed by this method. All of the primers are listed in Table S1 in Supplementary Material.

### Western Blotting

The total protein content was isolated from different follicles using Cell Lysis Reagent (Fermentas) and the protein concentration was determined using the bicinchoninic acid assay. Goat anti-mouse Wnt4 polyclonal antibody was purchased from Santa Cruz Biotechnology, Inc. Equal amounts of protein were separated by 10% sodium dodecyl sulfate gel electrophoresis under denaturing and non-reducing conditions and then transferred to polyvinylidene fluoride (PVDF) membranes. The PVDF membranes were first blocked, then incubated (1 h, 37°C) with the Wnt4 antibody diluted (1:500) in a 5% bovine serum albumin (BSA)/PBS solution. After washing in phosphate-buffered saline with Tween 20, the blots were incubated with horseradish-peroxidase-conjugated goat anti-mouse immunoglobulin G antibody (1:1,000; Abcam) in a 5% BSA/PBS solution. The signals were visualized using a 3,3′-diaminobenzidine (DAB) substrate kit (Tiangen).

### ELISA

The Wnt4 levels in the medium were detected using a Chicken Wingless-Type MMTV Integration Site Family Member 4 (Wnt4) ELISA Kit (Shanghai Enzyme-linked Biotechnology Co., Ltd.). The culture media of four kinds of cells [GCs and theca cells (TCs) of hierarchical follicles, and GCs and TCs of prehierarchical follicles] were collected and centrifuged at 3,000 rpm for 30 min to remove debris. The Wnt4 level was then determined according to the manufacturer’s instructions. The optical density of every well was tested at 450 nm using an ELx808 Absorbance Reader (BioTek, Winooski, VT, USA). The sample concentrations were calculated according to the mean absorbance.

### Cell Proliferation Assay

The proliferation of GCs was detected using an Enhanced Cell Counting Kit-8 Assay Kit (Beyotime, Beijing, China). Approximately 6 × 10^3^ cells were seeded in every well of a 96-well plate. The cells were transfected with the pUSEamp-Wnt4 plasmid and shRNA of *Wnt4* when the cells reached 60% confluence. At 0, 24, 48, and 72 h after transfection, 100 µL of medium with 10 µL of CCK8 was added to each well, and then the plates were incubated for a further 2 h at 38°C. The absorbance was evaluated using an ELx808 Absorbance Reader at 450 nm.

### Statistical Analyses

All data are presented as the mean ± SEM. The differences between different groups were determined by one-way ANOVA followed by Duncan’s test using the SPSS software (SPSS Inc., Chicago, IL, USA). The differences between groups were considered significantly different when *p* < 0.05.

## Results

### Sequencing Results Summary

RNA-seq was used to compare the transcriptomes of six small yellow follicles, which are referred to here as T1, T2, T3, T4, T5, and T6 (T1 and T4, T2 and T5, and T3 and T6 were derived from three different laying hens). The mRNA expression levels of *FSHR* for follicles T1, T4, T2, T5, T3, and T6 were 1.08 ± 0.01, 5.75 ± 0.27, 1.00 ± 0.06, 1.25 ± 0.16, 1.04 ± 0.19, and 1.08 ± 0.02, respectively, as determined by qRT-PCR, and the high-throughput RNA-seq generated 45.39, 44.14, 44.09, 43.81, 43.48, and 42.73 million raw reads, respectively, for the six follicles. Of these, 75.13–78.40% could be mapped to the chicken genome, and more than 98% of the reads were unique (Table 1). The sequencing reads were submitted to NCBI’s GEO under the accession number GSE100673.

**Table 1:** Summary of the RNA-seq results for 6–8 mm follicles.

Follicles	Total reads	Mapped reads	Unique mapped reads
T1	45,386,126	34,531,801 (76.08%)	33,958,795 (98.34%)
T2	44,094,300	33,394,769 (75.73%)	32,776,351 (98.15%)
T3	43,484,988	33,045,809 (75.99%)	32,479,464 (98.29%)
T4	44,143,998	34,610,174 (78.40%)	33,947,343 (98.08%)
T5	43,809,214	32,912,529 (75.13%)	32,292,304 (98.12%)
T6	42,730,428	32,314,323 (75.62%)	31,706,047 (98.12%)

A total of 17,993 genes were examined for the six follicles, and the expression of all of these genes is listed in Table S3 in Supplementary Material. In the process of screening for DEGs between groups, a fold change ≥2 and false discovery rate (FDR) <0.01 were used as the criteria. In the T1 vs. T4 group, 1,968 DEGs were detected, including 561 upregulated genes and 1,407 downregulated genes. In the T2 vs. T5 group, 289 DEGs were detected, including 104 upregulated genes and 185 downregulated genes. In the T3 vs. T6 group, 590 DEGs were detected, including 225 upregulated genes and 365 downregulated genes (Table S4 in Supplementary Material). To validate the RNA-seq data, some DEGs were chosen and quantified by qRT-PCR. The results showed that the mRNA levels of these genes were similar to the sequencing data (Table 2).

**Table 2:** Relative mRNA expression of ten selected genes from the three groups for comparison between the RNA-seq and qRT-PCR results.

Gene	T1 vs. T4 (T4/T1)		T2 vs. T5 (T5/T2)		T3 vs. T6 (T6/T3)	
	qRT-PCR	RNA-seq	qRT-PCR	RNA-seq	qRT-PCR	RNA-seq
*PPARG*	2.78 ± 0.24	2.55	1.30 ± 0.06	1.23	2.06 ± 0.21	2.00
NM_001001460.1						

*PTHLH*	3.07 ± 0.11	2.66	0.92 ± 0.13	1.08	1.68 ± 0.31	1.66
NM_001174106.1						

*SMAD2*	2.71 ± 0.08	2.89	0.70 ± 0.07	1.36	1.85 ± 0.17	1.98
XM_001232180.4						

*Wnt4*	1.95 ± 0.21	2.12	−1.05 ± 0.20	−0.67	1.32 ± 0.17	2.10
NM_204783.1						

*Follicle-stimulating hormone receptor*	2.41 ± 0.09	1.99	0.30 ± 0.17	0.53	0.12 ± 0.28	1.00
NM_205079.1						

*FZD1*	0.17 ± 0.10	7.92	0.06 ± 0.12	0.94	−0.08 ± 0.07	1.17
NM_001030337.1						

*AXIN2*	0.02 ± 0.2	−0.98	−0.08 ± 0.09	−0.18	−0.62 ± 0.18	−0.57
NM_204491.1						

*WNT5A*	−0.81 ± 0.33	−1.37	−1.44 ± 0.39	−0.05	−2.08 ± 0.24	−0.24
NM_204887.1						

*WNT9A*	−2.36 ± 0.14	−2.20	−0.07 ± 0.06	0.41	−0.56 ± 0.05	−0.29
NM_204981.2						

*WIF1*	0.88 ± 0.07	−2.64	−0.35 ± 0.11	−0.49	−0.76 ± 0.07	−0.49
NM_001199607.2						

*qRT-PCR data are shown as mean of fold change ± SEM. RNA-seq data are shown as mean of fold change (false discovery rate)*.

### Functional Analysis of DEGs

**Figure 1:**
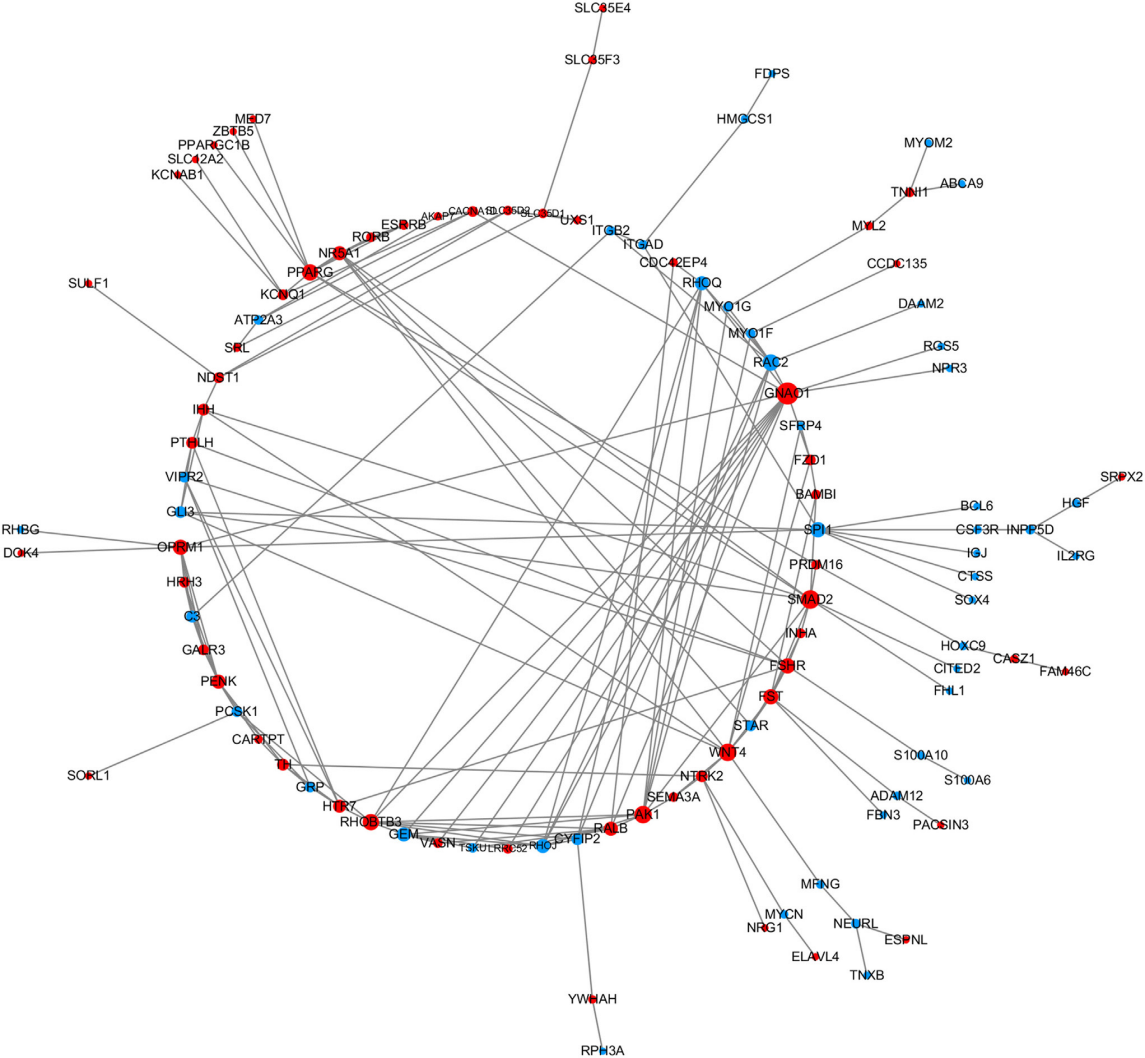
The protein–protein interaction network of the differentially expressed genes (DEGs). STRING was used to analyze the DEGs. The network nodes represent the genes shown in Table S5 in Supplementary Material (red and blue indicate upregulated and downregulated genes, respectively) and the lines represent interactions between genes.

In order to explore the key candidate genes related to follicle development, a network of the DEGs was obtained using STRING PPI network analysis (Figure 1); all of the genes are listed in Table S5 in Supplementary Material. Some of the genes, for instance *Smad2, Wnt4*, and *FSHR*, were found to play vital roles in the network.

In the three groups, with the increase in *FSHR* expression, the number of DEGs increased. To further explore the biological functions of these genes, we performed KEGG analysis and GO assignments of the DEGs, and the corresponding information is listed in Tables S6 and S7 in Supplementary Material. The significantly enriched KEGG pathways of the three groups are shown in Table 3. In the T1 vs. T4 group, the Wnt signal pathway was significantly enriched, and the DEGs of the Wnt signal pathway are shown in Table 4. In the follicle with the highest expression level of *FSHR*, the expression level of *Wnt4* was also significantly higher than the follicle with the lowest expression level of *FSHR*, with a log_2_(fold change) of 2.12. We hypothesized that *Wnt4* may play an important role in follicle selection and development, and therefore subsequently analyzed its function.

**Table 3:** Significantly enriched pathways.

Pathway	*p* Value	UniGene number
**T1 vs. T4**		

Focal adhesion	0	76
ECM–receptor interaction	6.80E−13	46
Cytokine–cytokine receptor interaction	1.42E−09	52
Cell adhesion molecules	1.13E−07	33
TGF-β signaling pathway	0.000100699	21
Vascular smooth muscle contraction	0.000111614	27
Amebiasis	0.000368349	6
Melanogenesis	0.000937358	22
Calcium signaling pathway	0.003524408	33
Adherens junction	0.004793109	17
MAPK signaling pathway	0.005027368	43
Regulation of actin cytoskeleton	0.006272819	36
Wnt signaling pathway	0.006320051	27
Gap junction	0.0071262	18
Steroid hormone biosynthesis	0.009703041	9
Arrhythmogenic right ventricular cardiomyopathy	0.013001616	5
VEGF signaling pathway	0.01314305	15
Gastric acid secretion	0.013974998	2
Intestinal immune network for IgA production	0.024274613	8
Phosphatidylinositol signaling system	0.028484703	16

**T2 vs. T5**		

PPAR signaling pathway	0.007689374	4
Focal adhesion	0.016837656	7
Progesterone-mediated oocyte maturation	0.019698938	4
Dorsoventral axis formation	0.03060988	2
Cardiac muscle contraction	0.035262584	3
Lipoic acid metabolism	0.039079049	1
Oocyte meiosis	0.040688919	4
Adipocytokine signaling pathway	0.047671798	3
Wnt signaling pathway	0.848944256	1

**T3 vs. T6**		

Steroid biosynthesis	0.0003184	5
Phagosome	0.0042824	12
NOD-like receptor signaling pathway	0.005853671	6
Terpenoid backbone biosynthesis	0.017505751	3
DNA replication	0.031551297	4
Glycosaminoglycan biosynthesis—chondroitin sulfate	0.048837386	3
Wnt signaling pathway	0.441815127	6

**Table 4:** Differentially expressed genes of Wnt signal pathway in the T1 vs. T4 group.

Gene	False discovery rate	Log_2_(fold change)	Up- or downregulated
*FZD1, Fz-1, cFz-1*	0	7.916035833	Up
*SMAD2, MADH2*	0	2.887762211	Up
*Wnt4*	6.66E−16	2.115271676	Up
*WIF1*	0.000361044	1.145351015	Up
Similar to strabismus	0.006074629	−1.050277449	Down
*DAAM2*	0.003280038	−1.089648489	Down
*PLCB1*	0.008015936	−1.104548086	Down
*NFATC2*	0.003303788	−1.106378948	Down
*SFRP4*	0.00029446	−1.158226013	Down
*SFRP1, CSFRP1*	4.51E−05	−1.237170155	Down
Similar to disheveled, dsh homolog 1	8.21E−05	−1.261713034	Down
*WNT5A*	0.000213247	−1.366774461	Down
*PRKCA*	2.29E−07	−1.526470318	Down
*CAMK2D*	1.17E−06	−1.531255044	Down
Similar to protein kinase C beta type	4.18E−05	−1.552007291	Down
*PRKCA*	1.69E−07	−1.566220886	Down
Hypothetical protein LOC100230090	0.000575417	−1.731836055	Down
*FZD3, FZ-3, cFz-3*	3.83E−05	−1.873285715	Down
*PRICKLE2*	3.48E−11	−2.005821446	Down
Similar to HMG-box transcription factor TCF-3	7.66E−10	−2.014370297	Down
*LEF1, LEF-1*	1.78E−09	−2.026783917	Down
*NFATC1*	1.01E−05	−2.057215317	Down
*FZD2, cFz-2*	1.51E−12	−2.073360036	Down
*WNT5B*	8.38E−12	−2.098749215	Down
*WNT9A, WNT-14*	5.76E−12	−2.200244472	Down
*SFRP5*	4.11E−13	−2.606370027	Down
*FZD7, Fz-7, cFz-7*	1.36E−11	−3.265319335	Down

### Expression Characteristics of *Wnt4* in Follicles

**Figure 2:**
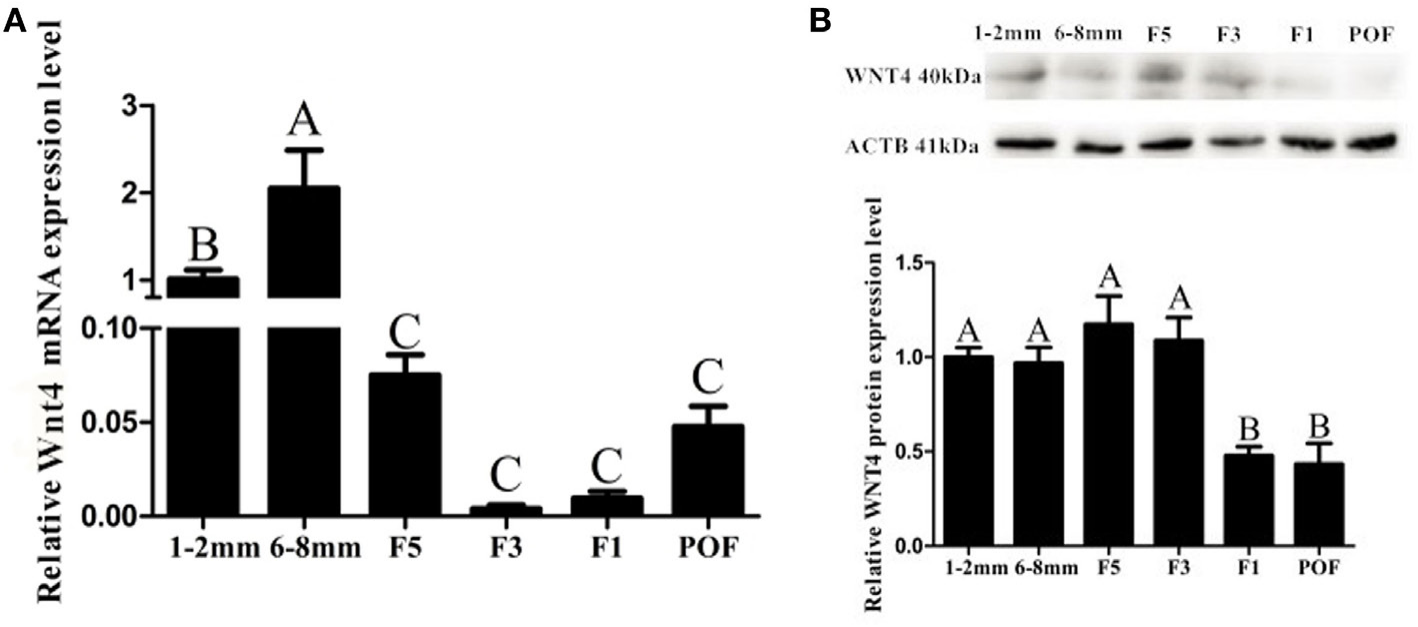
Expression of *Wnt4* in follicles at different developmental stages from 30-week-old hens. **(A)** Expression levels of *Wnt4* mRNA in 1–2 mm follicles, 6–8 mm follicles (small yellow follicles), the fifth largest follicles (F5), the third largest follicles (F3), the largest follicles (F1), and the newly postovulatory follicles (POFs). **(B)** Expression levels of Wnt4 protein in the various follicles. Data are presented as the mean ± SEM from at least three independent experiments. Bars with different letters are significantly different (^ABC^*p* < 0.01).

**Figure 3:**
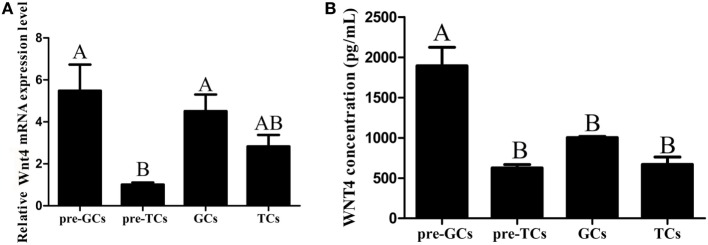
Expression of *Wnt4* in granulosa cells (GCs) and theca cells (TCs). **(A)** mRNA expression of chicken *Wnt4* in the GCs and TCs of hierarchical follicles and the GCs (pre-GCs) and theca cells (pre-TCs) of prehierarchical follicles. **(B)** Protein expression of chicken Wnt4 in GCs, TCs, pre-GCs, and pre-TCs (^ABC^*p* < 0.01).

*Wnt4* was expressed in both the prehierarchical and hierarchical follicles, but the expression level in the prehierarchical follicles was significantly higher than that in the hierarchical follicles (*p* < 0.01). The *Wnt4* expression level was found to be highest in the small yellow follicles (6–8 mm), then declined significantly in the F5 follicles (*p* < 0.01), and this downward trend continued until F1, then slightly increased in the postovulatory follicles (POFs) (Figure 2A). On the protein level, Wnt4 was highly expressed in the prehierarchical, F5, and F3 follicles, but declined significantly in the F1 follicles and POFs (*p* < 0.01) (Figure 2B). In both the hierarchical and prehierarchical follicles, the *Wnt4* mRNA and protein levels were significantly higher in the GCs than in the TCs (*p* < 0.01) (Figure 3).

### Effect of *Wnt4* on Steroidogenic Enzyme Gene Expression in GCs

**Figure 4:**
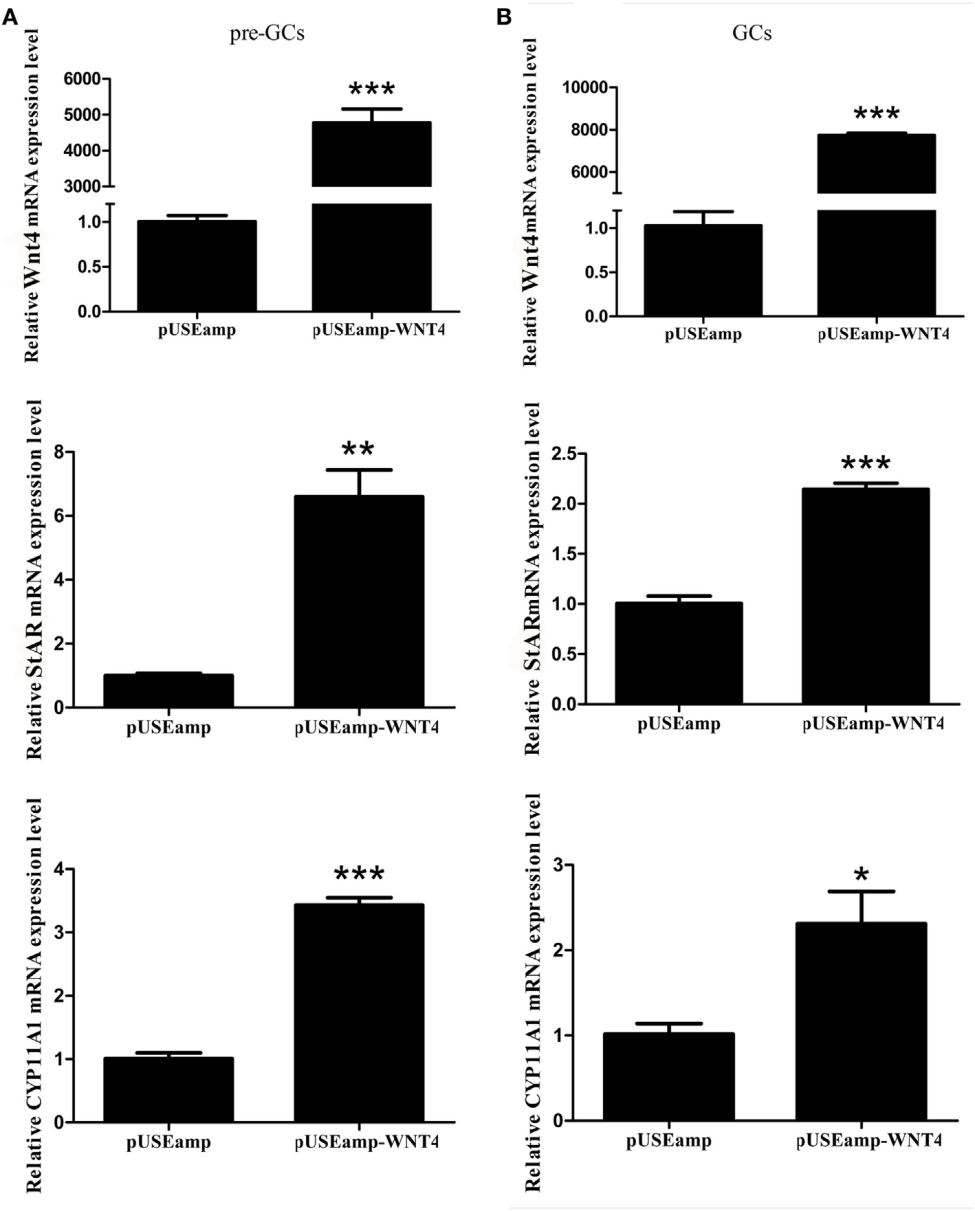
Effect of *Wnt4* overexpression on the expression of *StAR* and *CYP11A1* in **(A)** pre-GCs and **(B)** GCs of chicken follicles (*p < 0.05, **p < 0.01, ***p ≤ 0.001).

**Figure 5:**
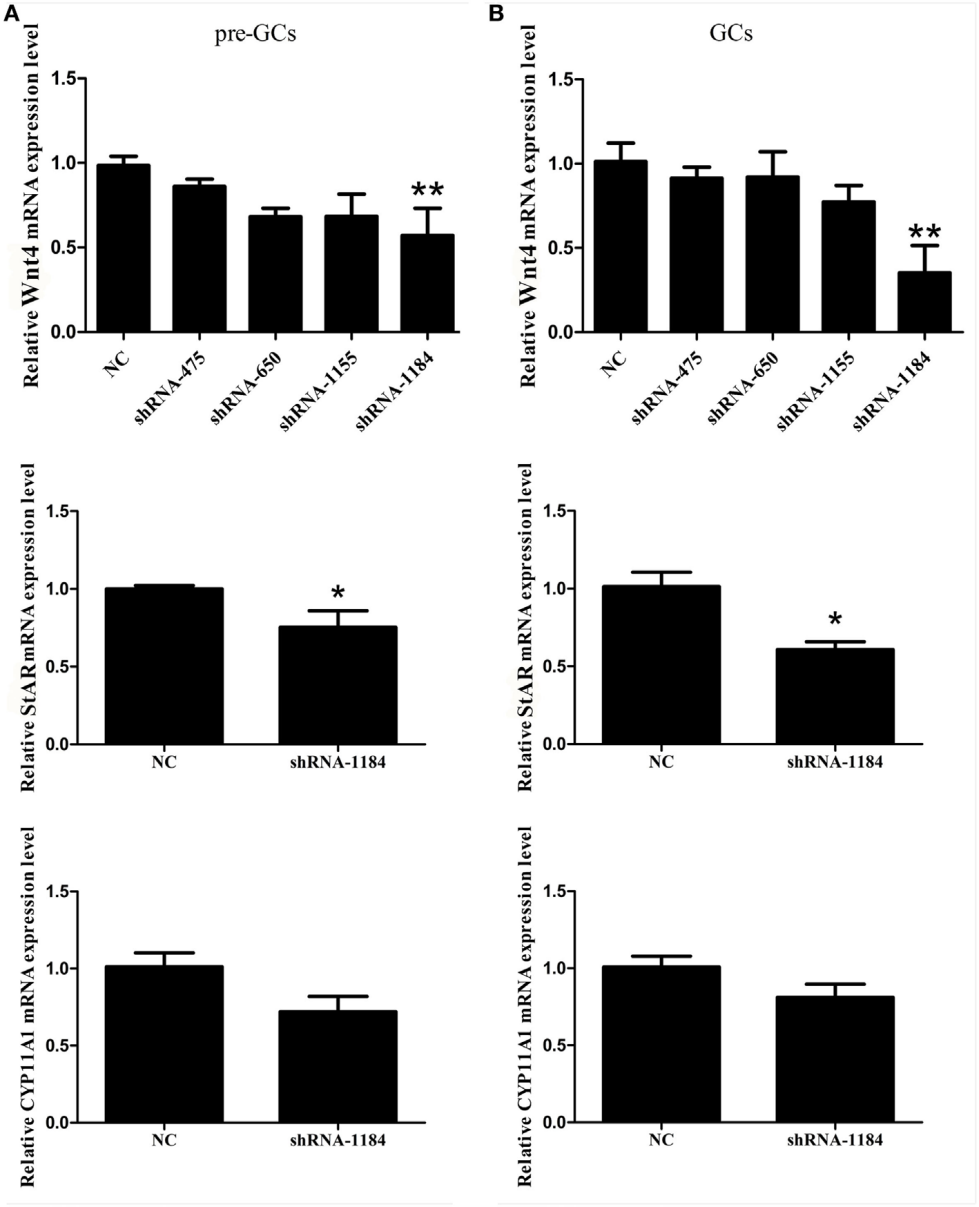
Effect of *Wnt4* knockdown on the expression of *StAR* and *CYP11A1* in **(A)** pre-GCs and **(B)** GCs of chicken follicles (*p < 0.05, **p < 0.01).

To determine the effect of *Wnt4* on steroidogenic enzyme gene expression, GCs were treated with *Wnt4* overexpression vectors and shRNA. In pre-GCs, the expression levels of *StAR* (*p* < 0.01) and *CYP11A1* (*p* ≤ 0.001) mRNA were significantly increased as a result of *Wnt4* overexpression (Figure 4A). In GCs, the expression levels of *StAR* (*p* ≤ 0.001) and *CYP11A1* (*p* ≤ 0.05) were also significantly increased (Figure 4B). The mRNA expression levels of *StAR* (*p* < 0.05) were significantly reduced in pre-GCs and GCs after knockdown of *Wnt4* expression, whereas those of *CYP11A1* were not significantly reduced (Figure 5).

### Effect of *Wnt4* on the Proliferation of GCs

**Figure 6:**
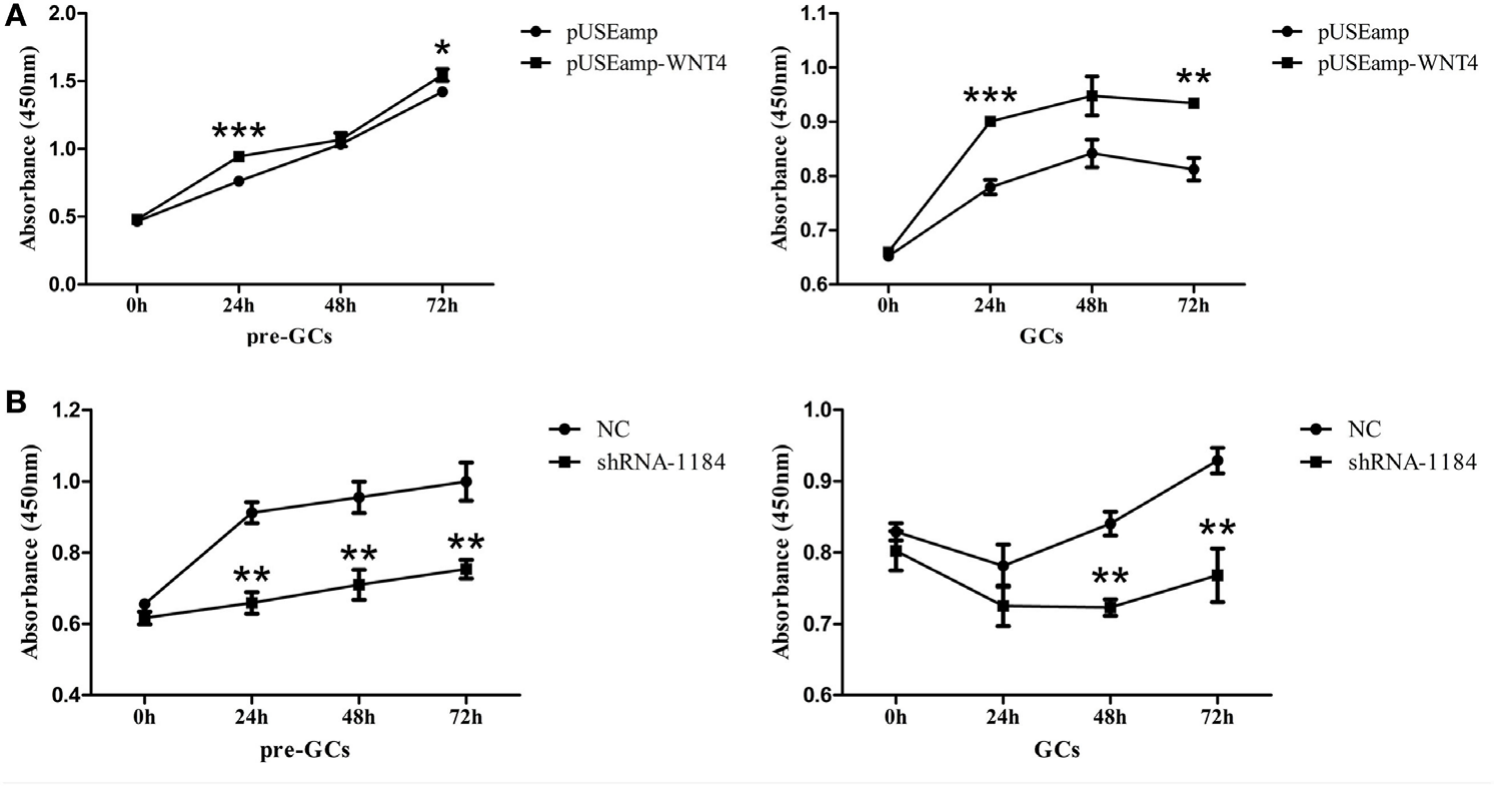
Effect of *Wnt4* on chicken granulosa cell (GC) proliferation. **(A)** Overexpression of *Wnt4* in GCs stimulated cell proliferation, as determined by the CCK8 method. **(B)** Knockdown of *Wnt4* in GCs inhibited cell proliferation, as determined by the CCK8 method. (*p < 0.05, **p < 0.01, ***p ≤ 0.001).

As *Wnt4* was highly expressed in GCs, we speculated that it is likely involved in GC proliferation in fast-growing follicles. Consequently, GCs were transfected with the overexpression vector pUSEamp-Wnt4 and the shRNA of *Wnt4* to examine the effect of *Wnt4* on their proliferation. We found that the overexpression of *Wnt4* significantly stimulated the proliferation of GCs (*p* < 0.05) (Figure 6A). Conversely, when the *Wnt4* expression level was knocked down, the proliferation of GCs significantly decreased (*p* < 0.01) (Figure 6B).

### Involvement of *Wnt4* in Follicle Selection

**Figure 7:**
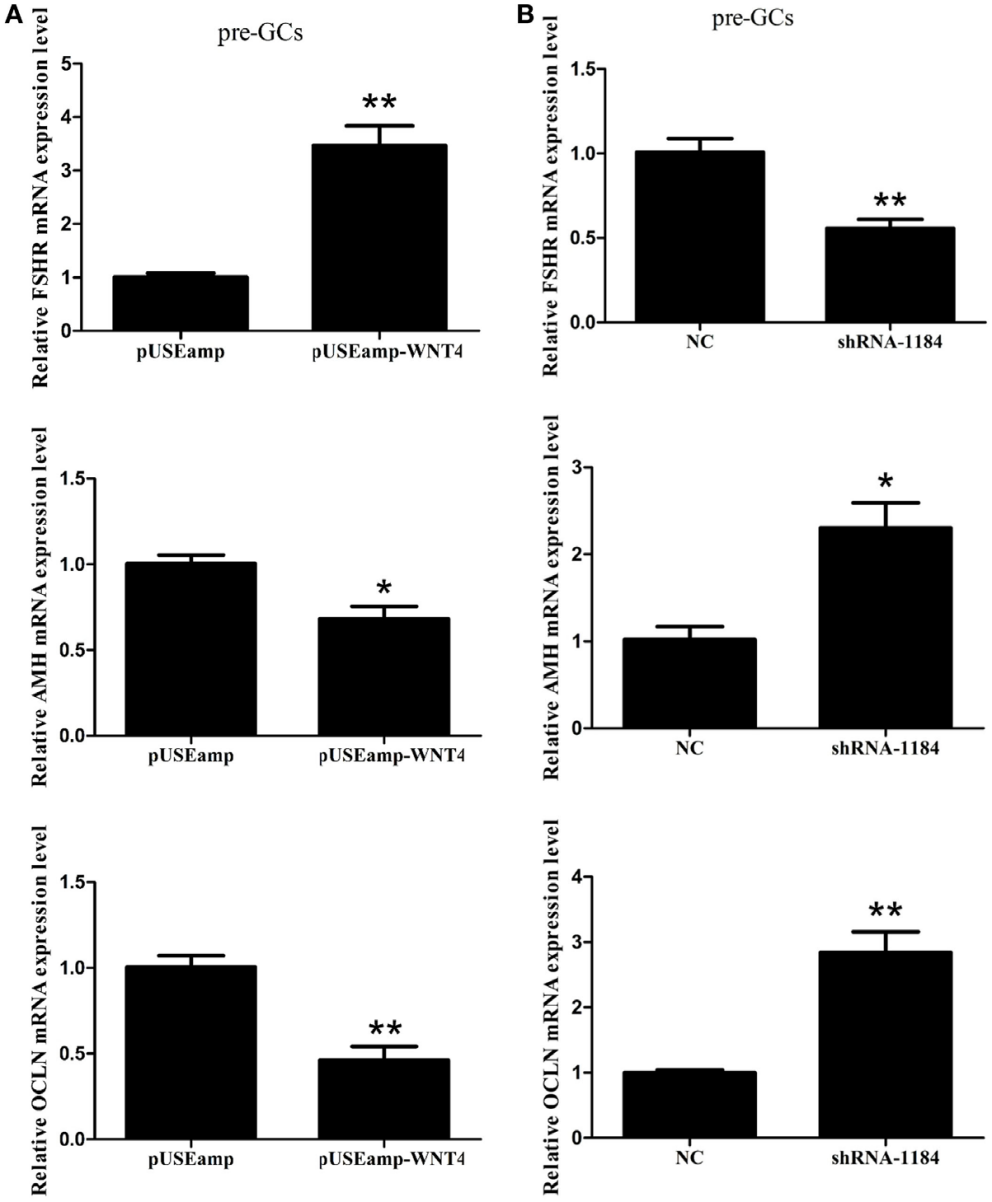
Effect of *Wnt4* on follicle-stimulating hormone receptor (*FSHR*), anti-Müllerian hormone (*AMH*), and *OCLN* expression in prehierarchical follicle granulosa cells (GCs). **(A)** Overexpression of *Wnt4* increased the expression level of *FSHR*, but decreased those of *AMH* and *OCLN*. **(B)** Knockdown of *Wnt4* decreased the expression level of *FSHR*, but increased those of *AMH* and *OCLN* (*p < 0.05, **p < 0.01).

*Follicle-stimulating hormone receptor, AMH*, and *OCLN* are the key regulators of follicle selection, and so we determined the expression levels of these genes upon changing the expression level of *Wnt4*. In the prehierarchical GCs, the expression level of the mRNA for *FSHR* (*p* < 0.01) was significantly increased by the overexpression of *Wnt4*, and the expression levels of *AMH* (*p* < 0.05) and *OCLN* (*p* < 0.01) were simultaneously decreased (Figure 7A). Furthermore, the expression levels of *AMH* (*p* < 0.05) and *OCLN* (*p* < 0.01) mRNA were increased in prehierarchical follicle GCs treated with the shRNA of *Wnt4*, and the level of *FSHR* mRNA (*p* < 0.01) was simultaneously decreased (Figure 7B).

### Effect of FSH on *Wnt4* Expression

**Figure 8:**
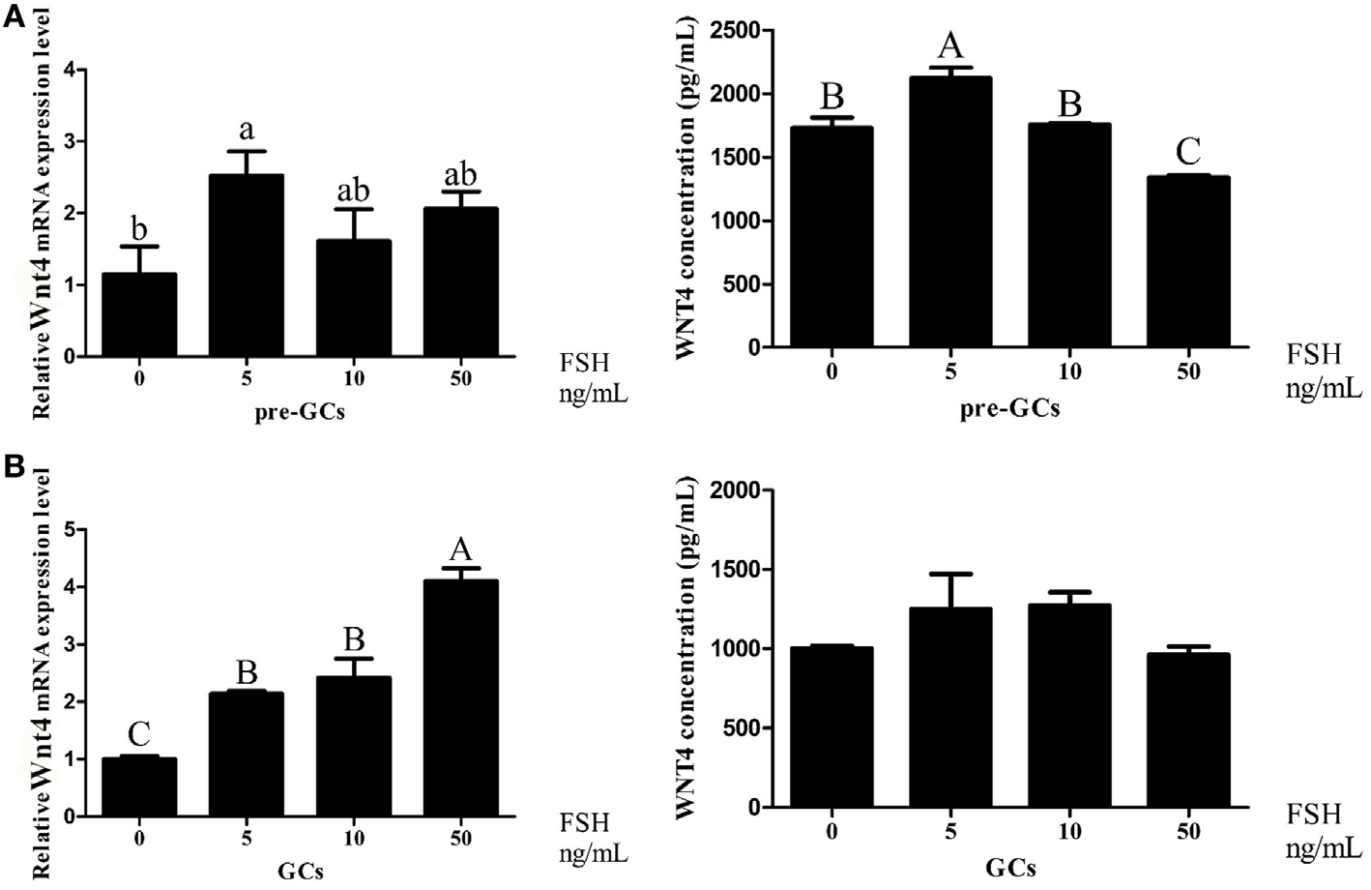
Effects of follicle-stimulating hormone (FSH) treatment on the *Wnt4* mRNA and protein levels in the granulosa cells (GCs) of ovarian follicles. Various doses of FSH (0, 5, 10, and 50 ng/mL) were tested. **(A)** Effect of FSH on *Wnt4* in the GCs of prehierarchical follicles. **(B)** Effect of FSH on *Wnt4* in the GCs of hierarchical follicles. All data are presented as the mean ± SEM. (^abc^*p* < 0.05; ^ABC^*p* < 0.01).

Follicle-stimulating hormone treatment at 5 ng/mL increased the expression of *Wnt4* at both the mRNA (*p* < 0.05) and protein (*p* < 0.01) levels in the GCs of prehierarchical follicles (Figure 8A). In hierarchical follicles, with the increase of FSH concentration, the expression level of *Wnt4* gradually increased (*p* < 0.01). Although a slight upward trend seems to occur, no significant difference of Wnt4 protein levels was observed in the hierarchical follicles (Figure 8B).

### Suppression of the Effects of FSH on Steroidogenic Enzyme Gene Expression in GCs of Prehierarchical Follicles by *Wnt4*

**Figure 9:**
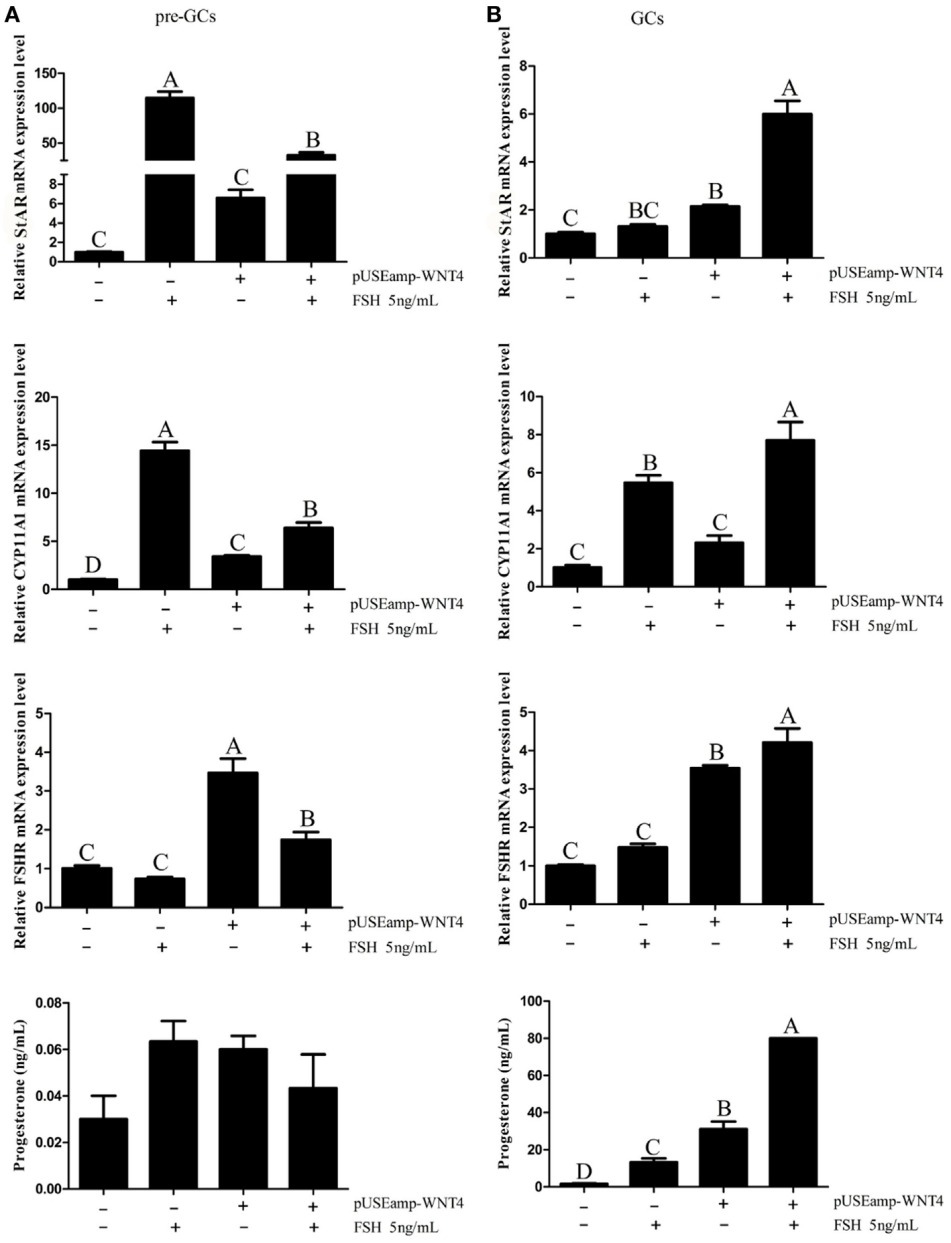
Effects of *Wnt4* and follicle-stimulating hormone (FSH) on mRNA expression of steroidogenic enzyme genes and steroidogenesis in prehierarchical and hierarchical follicles. **(A)** Effects of *Wnt4* and FSH on *StAR, CYP11A1*, and follicle-stimulating hormone receptor (*FSHR*) mRNA expression and progesterone secretion in the granulosa cells (GCs) of prehierarchical follicles. Bars with different superscript letters are significantly different. **(B)** Effects of *Wnt4* and FSH on *StAR, CYP11A1*, and *FSHR* mRNA expression and progesterone secretion in the GCs of hierarchical follicles. (^ABC^*p* < 0.01).

As the *Wnt4* expression level changed during follicle development and was affected by the concentration of FSH, we further focused on the function of *Wnt4* in follicular selection and development. In the GCs of prehierarchical follicles, FSH significantly increased the mRNA expression level of *StAR* and *CYP11A1* (*p* < 0.01), although it had no significant stimulatory effect on progesterone and no effect on the expression of *FSHR*. When GCs were treated with a combination of FSH and *Wnt4, Wnt4* suppressed the effect of FSH on the *StAR* and *CYP11A1* mRNA levels (*p* < 0.01) (Figure 9A).

### Facilitation of the Effects of FSH on Steroid Secretion and Steroidogenic Enzyme Gene Expression in GCs of Hierarchical Follicles by *Wnt4*

In the GCs of hierarchical follicles, FSH significantly increased the mRNA expression level of *FSHR* and the concentration of progesterone (*p* < 0.01), but not those of *StAR* and *CYP11A1*. When GCs were treated with a combination of FSH and *Wnt4*, progesterone secretion and steroidogenic gene expression were both significantly increased (*p* < 0.01) to levels exceeding those observed upon treatment with FSH or *Wnt4* alone (Figure 9B).

## Discussion

Follicular selection and development are important for egg production and laying performance in the poultry industry. Understanding gene expression patterns during the follicle selection period is crucial to understanding the laying mechanism and improving the laying performance of hens. Recent studies have shown that all of the small yellow follicles isolated from the same ovary in a laying hen can express FSHR and respond to stimulation by FSH (25–27), and one of them contains significantly higher *FSHR* mRNA expression levels than the others, and this follicle will be selected to the hierarchical follicles (14). In this study, follicles with different *FSHR* expression levels displayed different transcriptomes and the Wnt signal pathway was significantly enriched along with the higher expression of the *FSHR* gene. Consequently, we investigated the DEGs during the period of follicle selection and the role of *Wnt4* in follicle selection and development.

Follicle selection, growth, and muturation depend upon many endocrine, paracrine, and autocrine signals. The selection of one follicle from a large pool of small yellow follicles to form a hierarchical follicle requires the participation of signal molecules. The evidence has shown that during the period of follicle selection, the TGF-β signaling pathway is involved in initiating follicle selection (7, 8, 11). *BMP15* may promote follicle selection in hens; with the production of *BMP15*, the oocyte regulates the surrounding somatic cells during follicle selection (9). Moreover, *BMP6* was found to promote *FSHR* and *AMH* expression in the GCs from prehierarchical follicles in hens, and FSH signaling suppresses *AMH* expression and initiates the differentiation of GCs within the selected follicles (3). In our previous study, the gene expression profiles of small white follicles, F1, and POF were obtained by RNA-seq (28). In this study, DEGs were identified by the comparison of small yellow follicles expressing significantly different levels of *FSHR* from the same laying hens, to explore the molecular mechanisms of follicle selection. Hundreds of DEGs and tens of signal pathways, including the TGF-β and Wnt signaling pathways, were revealed. The expression of *Wnt4* was significantly increased in the small yellow follicles of laying hens with the highest expression of *FSHR*, suggesting that *Wnt4* is likely to be involved in follicle selection and changes in its expression level may be related to the effect of FSH. Therefore, the role of *Wnt4* in follicle selection is subsequently investigated.

Previous studies have unveiled the contributions of members of the Wnt family to follicular development and steroid production in rodents (29, 30). *Wnt4* was expressed in small avian follicles (0.5–2 mm) and was downregulated during early follicular development (31). *Wnt4* is known for regulating differentiation of the embryonic ovary (32) and is highly expressed in small mammalian follicles. In this study, *Wnt4* was found to be expressed in follicles of all sizes in hens. *Wnt4* was more highly expressed in the small yellow follicles than in the 1–2 mm follicles, and in chicken follicles, the expression of *Wnt4* was significantly higher in the GCs than in the TCs, which is in accordance with the results obtained in rats, mice, and humans (33–36). In mice, *Wnt4* was expressed in GCs throughout follicle development (21).

When chicken GCs were treated with the overexpression vector and shRNA of *Wnt4*, the expression levels of *CYP11A1* and *StAR* were greatly altered. Chicken GC differentiation involves the responsiveness of GC layers to FSH, which is measured by their capacity to produce steroids such as *StAR* and progesterone (37). The overexpression of *Wnt4* in mice GCs resulted in the increased expression of *CYP11A1* and *StAR*. *Wnt4*-null mice displayed lower expression levels of *CYP11A1* and *StAR* compared to the controls (20). These results are consistent with ours, suggesting that *Wnt4* likely stimulated GC differentiation. Moreover, under the same conditions, we found that the overexpression of *Wnt4* stimulated the cell proliferation of GCs from both prehierarchical and hierarchical follicles, whereas the knockdown of *Wnt4* expression produced the opposite effect, suggesting that *Wnt4* could stimulate GC proliferation.

Both the viability of chicken prehierarchical follicles and the cell differentiation associated with the selection of a single small yellow follicle per day into the hierarchical follicles depend on FSH and the expression level of *FSHR* in GCs. *FSHR* is expressed in GCs at all stages of prehierarchical follicles, but only one small yellow follicle can express the highest *FSHR* mRNA level, and this follicle will be recruited to become a hierarchical follicle (14). *AMH* is a member of the TGF-β family, and its mRNA expression level declines as the follicle size decreases; it is most abundant in 1–2 mm follicles (11) and significantly decreases during the period of follicle selection (2). Tight junction proteins including *OCLN* are present in GCs. It has been suggested that *OCLN* could regulate access of the yolk to the oocyte surface around the time of follicle selection (12). The expression levels of *FSHR, AMH*, and *OCLN* changed after follicle selection (2, 11, 12). We speculated that *Wnt4* could participate in follicle selection by regulating the expression levels of these genes. In the present study, *Wnt4* increased the expression level of *FSHR*, but decreased the mRNA levels of *AMH* and *OCLN*. Furthermore, when the expression level of *Wnt4* declined, the expression of these genes increased. The effects of *Wnt4* on the expression of *FSHR, AMH*, and *OCLN* suggest that *Wnt4* plays a role in follicle selection in hens.

Evidence suggests that the expression of Wnt family genes is hormonally regulated in rodent and bovine ovaries. In rodent ovaries, *Wnt4* expression is detected in response to human chorionic gonadotropin (21), and in bovine GCs, upregulation of *Wnt2* mRNA expression was elevated when treated with FSH (38). During the period of chicken follicle selection, the small yellow follicle expressing the highest *FSHR* level acquires optimal responsiveness to FSH (1). As for the regulation of Wnt4 expression by FSH in chicken follicles, we found that *Wnt4* expression is elevated in GCs following FSH stimulation. We further analyzed the combinatorial effect of *Wnt4* and FSH on chicken prehierarchical and hierarchical follicles. In the GCs of hierarchical follicles, *Wnt4* can strengthen the influence of FSH; however, in prehierarchical follicles, *Wnt4* has an inhibitory effect on the function of FSH. Several studies have suggested that FSH and Wnt signaling pathways may work together to influence steroid production in the postnatal ovary, as in the case of humans, rats, and cows (39–41). Johnson supposed that the responsiveness to FSH was suppressed in prehierarchical follicles, but in hierarchical follicles, some signals are activated, the follicles become responsive to FSH (1), and the responsiveness to FSH is initiated by increasing cAMP production in GCs (3). In the GCs of prehierarchical follicles, there may exist an inhibitory or non-activated signal, and the GCs cannot respond to FSH (1). The difference in the effect of *Wnt4* on the action of FSH may suggest an interaction of Wnt4 signaling and FSH signaling in hierarchical follicles, but in prehierarchical follicles, as the small yellow follicle is not selected and GCs are not responsive to FSH, the action of FSH is not further strengthened. Similarly, during bovine dominant follicle selection, Wnt signaling was able to potentiate the action of FSH (42). Based on this result, we suppose that during follicle selection, along with the increased expression of *FSHR*, one small yellow follicle expresses more *Wnt4*, and the *Wnt4* subsequently stimulates the expression of *FSHR* to enhance the action of FSH signaling, and as such the *Wnt4* and FSH pathways act in combination during the development of the follicle from the prehierarchical stage to the hierarchical stage.

In conclusion, transcriptome analysis of single small yellow follicles expressing different levels of *FSHR* mRNA revealed a role of *Wnt4* in chicken follicle selection. The expression of *Wnt4* was significantly increased in small yellow follicles, and was higher in GCs than in TCs. *Wnt4* could increase the expression of *StAR, CYP11A1*, and *FSHR* mRNA in the GCs of prehierarchical and hierarchical follicles and promote GC proliferation. *Wnt4* decreased the expression of *AMH* and *OCLN*, and its expression was stimulated by FSH. The interaction of *Wnt4* and FSH signaling was strengthened from the prehierarchical to hierarchical follicles during the process of follicle selection. This study revealed for the first time that *Wnt4* stimulates follicle selection by enhancing *FSHR* expression and GC proliferation and steroidogenesis.

## Ethics Statement

All of the animal experiments were approved by the Institutional Animal Care and Use Ethics Committee of Shandong Agricultural University and performed in accordance with the “Guidelines for Experimental Animals” of the Ministry of Science and Technology of China.

## Author Contributions

YW and YJ contributed to the overall design of the manuscript. YW collected data. QC, ZL, XG, MG, ZY, and YD have contributed by sampling. YW, YJ, YS, and LK have participated in manuscript writing and revision. All authors approved the final version of the manuscript.

## Conflict of Interest Statement

The authors declare that the research was conducted in the absence of any commercial or financial relationships that could be construed as a potential conflict of interest.

## Supplementary Material

Table S1 Primers used in this study (DOCX).

Table S2 The target sequences of shRNA expression vectors (DOCX).

Table S3 Raw data of RNA-seq analysis (XLSX).

Table S4 The differentially expressed genes in six follicles (XLSX).

Table S5 Genes involved in protein–protein interaction network analysis (XLSX).

Table S6 KEGG pathways of differentially expressed genes (XLSX).

Table S7 Gene ontological classification of differentially expressed genes (XLSX).
