# Supplementary material for: Transcriptome Analysis on Single Small Yellow Follicles Reveals That Wnt4 Is Involved in Chicken Follicle Selection
Source: Front Endocrinol (Lausanne). 2017 Nov 15;8:317. doi: 10.3389/fendo.2017.00317 (PMC5694752; doi:10.3389/fendo.2017.00317)
Supplement: Table S1 — Primers used in this study (DOCX). [file table_1.docx]

TABLE S1 The primers used in the experiments

| Experiments | Gene Symbol | Accession Number (GenBank) | Primer Sequence | Product Size |
| --- | --- | --- | --- | --- |
| Real-time PCR | *WNT4* | NM_204783.1 | Forward: 5’ CCTGTCTTTGGCAAGGTGG  Reverse:5’ CATAGGCAATGTTATCGGAGC | 199bp |
|  | *FSHR* | NM_205079.1 | Forward: 5’ TTCCAGCCTTCCCAAACTA  Reverse:5’ GCCGTAGAATCACACTTTCAG | 258bp |
|  | *STAR* | NM_204686 | Forward: 5’ TGCCTGAGCAGCAGGGATTTATCA  Reverse:5’ TGGTTGATGATGGTCTTTGGCAGC | 149bp |
|  | *CYP11A* | NM_001001756 | Forward: 5’ ACTTCAAGGGACTGAGCTTTGGGT  Reverse:5’ AGTTCTCCAGGATGTGCATGAGGA | 103bp |
|  | *PPARG* | NM_001001460.1 | Forward: 5’ TCCTTCCCGCTGACCAAA  Reverse:5’ TCCTGCACTGCCTCCACA | 212bp |
|  | *PTHLH* | NM_001174106 | Forward: 5’ AGAGGAACTGCGACGAACA  Reverse:5’ GGATTGATTTGCCCTTGTC | 215bp |
|  | *SMAD2* | XM_001232180.4 | Forward: 5’ TGAGCTTGAGAAGGCCATTA  Reverse:5’ GCGCCACAGACGACAGTAGA | 223bp |
|  | *FZD1* | NM_001030337.1 | Forward: 5’ CCTGTTCATCGGCACCTCAT  Reverse:5’ GCTCTTACAGCTCTGCGTGA | 235bp |
|  | *AXIN2* | NM_204491.1 | Forward: 5’ AAAACGCTGAGGGTTACGG  Reverse:5’ ATCCCATCTACACTGCTGTCTGTC | 209bp |
|  | *WNT5A* | NM_204887.1 | Forward: 5’ TGCCAGTTGTATCAGGACCAT  Reverse:5’ AGGTGAACGCCGTCTCCC | 169bp |
|  | *WNT9A* | NM_204981.2 | Forward: 5’ CAGTACCAGTTTCGCTTTGAGC  Reverse:5’ CACCACAGCCACCCCACT | 229bp |
|  | *WIF1* | NM_001199607.2 | Forward: 5’ TCAGTGGTTCAGGTTGGGTT  Reverse:5’ TCCTGGACACTCCGCTTG | 159bp |
|  | *ACTB* | K02173.1 | Forward: 5’ TGGATGATGATATTGCTGC  Reverse:5’ ATCTTCTCCATATCATCCC | 253bp |
| Overexpression | *WNT4* | NM_204783.1 | Forward: 5’ CCCAAGCTTGCTGACAGTCGGGCTGG  Reverse:5’ CGCGGATCCGCAGCGTCTGGGTCGTT | 1317bp |
