# Supplementary material for: Transcriptome Analysis on Single Small Yellow Follicles Reveals That Wnt4 Is Involved in Chicken Follicle Selection
Source: Front Endocrinol (Lausanne). 2017 Nov 15;8:317. doi: 10.3389/fendo.2017.00317 (PMC5694752; doi:10.3389/fendo.2017.00317)
Supplement: Table S2 — The target sequences of shRNA expression vectors (DOCX). [file table_2.docx]

TABLE S2 The target sequences of shRNA expression vectors

| Vectors | Target sequences | Target sites |
| --- | --- | --- |
| pGPU6/GFP/Neo-WNT4-gallus-475 | GTCTTTGGCAAGGTGGTAACA | WNT4-gallus-mRNA-475 |
| pGPU6/GFP/Neo-WNT4-gallus-650 | GCTCCGATAACATTGCCTATG | WNT4-gallus-mRNA-650 |
| pGPU6/GFP/Neo-WNT4-gallus-1155 | GGAAAGGTGCAGCTGCAAATT | WNT4-gallus-mRNA-1155 |
| pGPU6/GFP/Neo-WNT4-gallus-1184 | GCTGCTCTGTCAAGTGCAAAC | WNT4-gallus-mRNA-1184 |
| pGPU6/GFP/Neo-WNT4-gallus-NC | GTTCTCCGAACGTGTCACGT |  |
